# The Effect of High-Dose Methyl Vitamin B12 Therapy on Epileptogenesis in Rats: An In Vivo Study

**DOI:** 10.7759/cureus.35929

**Published:** 2023-03-09

**Authors:** Aysha Fakhroo, Marya Al-Hammadi, Latifa Fakhroo, Fatima Al-Ali, Rania Snobar, Mohammed Al-Beltagi, Amer Kamal

**Affiliations:** 1 Department of Pediatrics, King Hamad University Hospital, Busaiteen, BHR; 2 Department of Anesthesia and Pain Management, King Hamad University Hospital, Busaiteen, BHR; 3 Department of General Practice, Royal College of Surgeons in Ireland, Busaiteen, BHR; 4 Department of Internal Medicine, King Hamad University Hospital, Busaiteen, BHR; 5 Department of Pediatrics, Al Qassimi Women’s and Children’s Hospital, Sharjah, ARE; 6 Department of Pediatrics, University Medical Center, King Abdulla Medical City, Arabian Gulf University, Dr. Sulaiman Al Habib Medical Group, Manama, BHR; 7 Department of Behavioral and Social Sciences, College of Science and Health, Webster University Leiden Campus, Leiden, NLD

**Keywords:** antiepileptic, seizure, vitamin b12, epilepsy, epileptogenesis

## Abstract

Introduction

Epileptogenesis has been considered one of the most prevalent diseases affecting significant numbers of individuals worldwide. Since vitamin B12 has been reported to possess antiepileptic effects, this supports that vitamin B12 deficiency is correlated to seizure occurrence. Hence, this study aimed to evaluate the neuroprotective effects of vitamin B12 injection on pentylenetetrazole (PTZ)-induced rats.

Methods

The study was performed using 40 adult female Sprague-Dawley rats (~250 g). A 45 mg/kg PTZ was intraperitoneally injected into rat models to induce seizure effects. Different groups of rat models received methyl vitamin B12 therapy at different dosages, a low dosage of 45 µg/kg and a high dosage of 85 µg/kg, at different pre-treatment periods, one day and two weeks prior to PTZ injection. A control group, which received only PTZ injection, served as a reference. The seizure latency, seizure intensity, and differences in the quality of seizures and their characteristics, from simple twitches to complete seizures, were observed after 30 minutes of PTZ injection.

Results

In general, the latency to convulsion significantly increased when vitamin B12 pre-treatment was employed. The longest latency time (LT) of 520.63±73.83 seconds was observed when a high dosage of vitamin B12 at 85 µg/kg was injected one day prior to PTZ inoculation, which was significantly higher than that of the control group at 176.88±62.67 seconds (P<0.001). Moreover, the duration of convulsion significantly decreased in which the lowest duration time (DT) of 7.00±4.68 seconds was observed when a high dosage of vitamin B12 at 85 µg/kg was injected two weeks prior to PTZ inoculation, which was significantly lower than that of the control group at 257.75±41.93 seconds (P<0.001). Lastly, the percentage of the population with PTZ-induced convulsion generally decreased after vitamin B12 pre-treatment in which majority showed more of simple less aggressive twitches rather than tonic-clonic seizures.

Conclusion

The results showed that vitamin B12 pre-treatment alleviates the seizure occurrence among PTZ-kindled rat models. These findings then suggest that vitamin B12 is a potential strategy and treatment for epilepsy and other related epileptogenesis activities.

## Introduction

Epileptogenesis is defined as molecular and cellular changes that consequently result in neuroinflammation, hypersynchrony, and hyperexcitability of cortical neurons [1,2]. Epilepsy is a chronic neurological state characterized by repetitive and unprovoked seizures [1,3-5]. Nearly 50 million people or 2% of the world population were diagnosed with epilepsy [3,5,6]. The prevalence rate of active epilepsy in most locations ranges from ﬁve to 10 cases per 1,000 population, as reported by Sander (2003) [4]. This disease can occur at all ages, and no constant age-speciﬁc incidence rate was observed; however, it was noted that this disease was less observed in younger ages compared to older age groups [4].

Since as early as the 1850s, epilepsy treatment involved different plant-based and animal-based extracts [7]. Nowadays, antiepileptic drugs (AEDs) have been extensively used as the major treatment for epilepsy; however, approximately 30%-40% of patients are resistant to these drugs [8-11]. Epilepsy is said to be drug-resistant when two AEDs given in appropriate doses fail to control seizure [5]. The ineffectiveness of AEDs and their possible side effects might contribute to some risks to the patients including neuronal damage. Although the effective drugs that can inhibit epileptogenesis are yet to be discovered, minimizing inflammatory reactions in the brain neurons can be a reasonable potential strategy against epilepsy [2]. Vitamins act as antioxidants and consequently can inhibit neuronal damage in the brain. Vitamin B12, also known as cobalamin, plays an important role in the formation of myelin sheaths in the central nervous system [12,13]. It is important in the methylation processes related to DNA and cell metabolism in which a deﬁciency may cause serious clinical impacts [14]. As an antioxidative and anti-inﬂammatory agent, vitamin B12 was observed to lessen oxidative damage and neuroinﬂammation, hence preventing epileptogenesis. As a matter of fact, vitamin B12 deﬁciency may result in a wide range of neurological, psychiatric, and hematologic consequences, including epileptic seizures [15]. Moreover, there are many studies, Erfanparast and Tamaddonfard (2015) [16] and Ikeda et al. (1997) [17], linking the antiepileptic effects of vitamin B12 to the enhancement of gamma-aminobutyric acid (GABA) activity.

In this study, the neuroprotective effect of vitamin B12 injection to pentylenetetrazol (PTZ)-induced seizure among rats has been evaluated.

## Materials and methods

Adult female Sprague-Dawley rats were used. The rats were housed in cages on sawdust and had access to food and water ad libitum. Animal care and experimental ethics were applied according to the guidelines of the research and animal ethics committee of Arabian Gulf University for the use of animals in experiments, under the reference number E015-PI-10/17. Vitamin B12 and PTZ were purchased from Sigma-Aldrich Co., St. Louis, MO, USA. The solutions were prepared on each day of the experiment.

Forty adult female Sprague-Dawley rats with an approximate weight of 250 g were used. Samples were divided into ﬁve groups comprising eight rats each. Seizure was induced via intraperitoneal injection of 45 mg/kg pentylenetetrazole (PTZ). Group 1, which received only PTZ injection, served as the control group. Groups 2 and 3 received methyl vitamin B12 therapy at a low dosage of 45 µg/kg and a high dosage of 85 µg/kg, respectively, one day prior to PTZ injection. While Groups 4 and 5 received methyl vitamin B12 therapy at a low dosage of 45 µg/kg and a high dosage of 85 µg/kg, respectively, two weeks prior to PTZ injection. The seizure latency, seizure intensity, and differences in the quality of seizures and their characteristics, from simple twitches to complete seizures, were observed after 30 minutes of PTZ injection. Video monitoring of animals was employed to ensure accurate time measurement and behavioral performance. All data were presented as mean±standard error of the mean (SEM) and analyzed using a one-tailed Student’s t-test. P values of less than 0.05 were considered statistically signiﬁcant. The data analyses were performed with Statistical Package for Social Sciences (SPSS) version 23 (IBM SPSS Statistics, Armonk, NY, USA).

## Results

**Figure 1:**
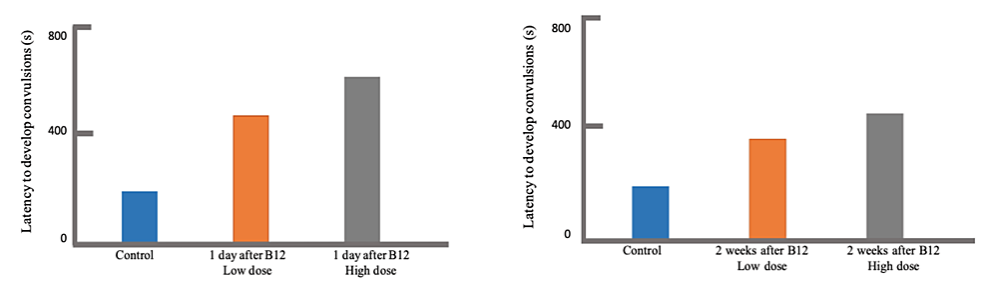
Comparison of latency to convulsion after vitamin B12 pre-treatment, one day (left) and two weeks (right), prior to PTZ injection. PTZ: pentylenetetrazole

The effects of vitamin B12 in the development of PTZ-induced seizure were demonstrated in terms of latency to convulsion (Table 1) and the duration of the convulsion (Table 2). The control group, which did not receive vitamin B12 treatment, had a latency mean±SEM of 176.88±62.67 seconds. In general, the latency to convulsion significantly increased when vitamin B12 was injected into the rat specimens. The longest latency time (LT) of 520.63±73.83 seconds was observed when a high dosage of vitamin B12 at 85 µg/kg was injected one day prior to PTZ inoculation (Group 3: N=8; P<0.001), while the shortest LT of 356.13±77.22 seconds was seen when a low dosage of vitamin B12 at 45 µg/kg was injected two weeks prior to PTZ inoculation (Group 4: N=8; P=0.047), which were still both significantly higher than that of the control group. From these, the highest obtained ΔLT was 421.00 seconds, while the lowest was 179.25 seconds. As shown in Figure 1, it was observed that the latency increases, in comparison to the control, as the dosage of vitamin B12 further increases from 45 µg/kg to 85 µg/kg (Group 2 versus Group 3; Group 4 versus Group 5). In addition, latency decreases as the time gap between vitamin B12 and PTZ injection widens from one day to two weeks (Group 2 versus Group 4; Group 3 versus Group 5).

**Table 1:** Comparison of latency to convulsions after PTZ administration. SEM, standard error of the mean; PTZ, pentylenetetrazole

Specimen number	Group 1	Group 2	Group 3	Group 4	Group 5
1	177	600	317	584	181
2	67	600	600	120	1,137
3	147	560	587	163	244
4	60	5	369	153	600
5	82	600	1,110	548	600
6	104	600	600	500	103
7	600	600	600	181	175
8	178	600	600	600	600
Mean, seconds	176.88	520.63	597.88	356.13	455.00
SEM, seconds	62.67	73.83	84.04	77.22	123.21
P value	---	0.002	<0.001	0.047	0.032

**Table 2:** Comparison of the duration of the PTZ-induced convulsion. SEM, standard error of the mean; PTZ, pentylenetetrazole

Specimen number	Group 1	Group 2	Group 3	Group 4	Group 5
1	261	79	5	12	7
2	300	223	23	30	1
3	304	413	38	0	6
4	400	45	88	68	0
5	97	74	2	122	0
6	100	0	0	34	39
7	200	0	44	569	3
8	400	81	0	0	0
Mean, seconds	257.75	114.38	25.00	104.38	7.00
SEM, seconds	41.93	49.27	10.92	67.92	4.68
P value	---	0.022	<0.001	0.038	<0.001

**Figure 2:**
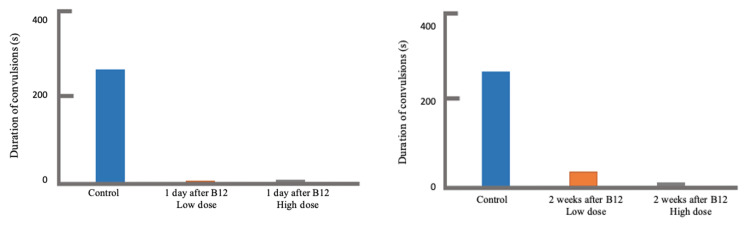
Comparison of convulsion duration after vitamin B12 pre-treatment, one day (left) and two weeks (right), prior to PTZ injection. PTZ: pentylenetetrazole

Moreover, the control group had a convulsion duration mean±SEM of 257.75±41.93 seconds. In general, the duration of convulsion significantly decreased when vitamin B12 was injected to the rat specimens. The lowest duration time (DT) of 7.00±4.68 seconds was observed when a high dosage of vitamin B12 at 85 µg/kg was injected two weeks prior to PTZ inoculation (Group 5: N=8; P<0.001), while the highest DT of 114.38±49.27 seconds was seen when a low dosage of vitamin B12 at 45 µg/kg was injected one day prior to PTZ inoculation (Group 2: N=8; P=0.022), which were still both significantly lower than that of the control group. From these, the highest obtained ΔDT was 250.75 seconds, while the lowest was 143.37 seconds. As presented in Figure 2, a shorter duration was observed as the dosage of vitamin B12 was increased from 45 µg/kg to 85 µg/kg (Group 2 versus Group 3; Group 4 versus Group 5). Consequently, the duration further shortens as the time gap between vitamin B12 and PTZ injection widens from one day to two weeks (Group 2 versus Group 4; Group 3 versus Group 5).

**Figure 3:**
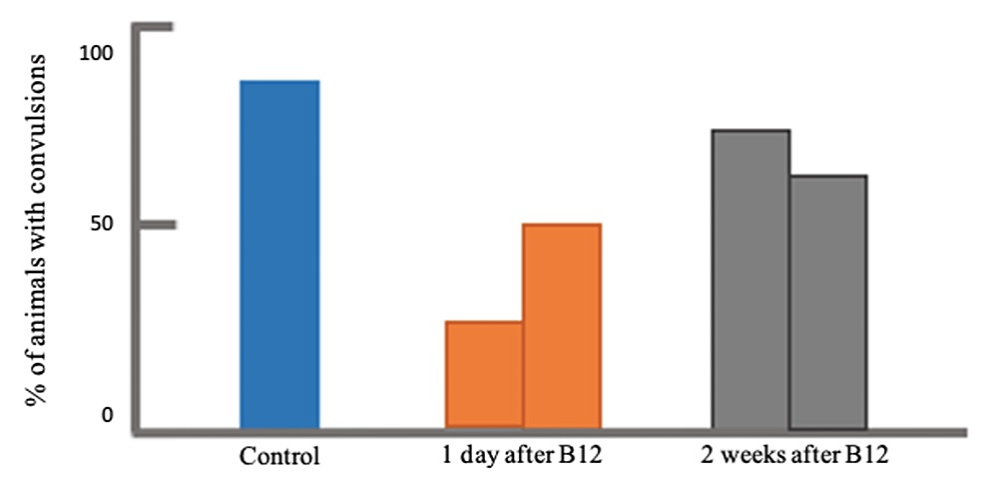
Comparison of the percentages of PTZ-induced convulsion development after vitamin B12 pre-treatment, one day and two weeks, prior to PTZ injection. PTZ: pentylenetetrazole

**Figure 4:**
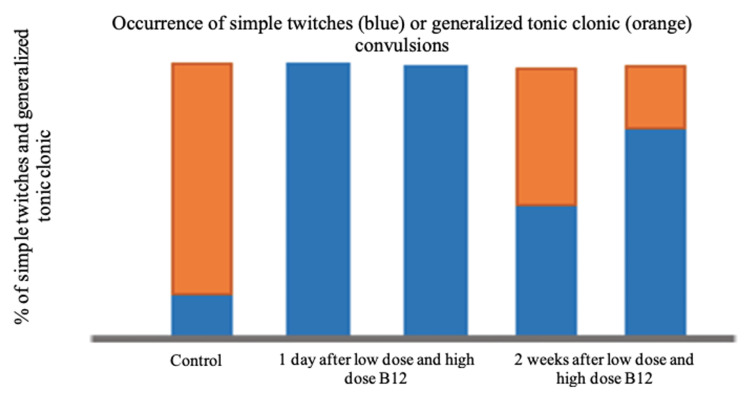
Comparison of the type of PTZ-induced convulsion occurring after vitamin B12 pre-treatment, one day and two weeks, prior to PTZ injection PTZ: pentylenetetrazole

The percentage of the population that developed the incidence of PTZ-induced convulsion was also evaluated. As shown in Figure 3, the lowest percentage was observed in the group that received a low dosage of vitamin B12 at 45 µg/kg, injected one day prior to PTZ inoculation. Although there was no general trend in terms of the effect of dosage and the time of vitamin injection, the percentage of the rats that developed convulsion generally decreased after vitamin B12 pre-treatment. Furthermore, the type of convulsion was also determined and was presented in Figure 4, either simple twitches (marked in blue) or tonic-clonic (marked in orange). The results revealed that majority of the specimens in the control group experienced a tonic-clonic convulsion rather than simple twitches. On the other hand, all samples under those groups that underwent vitamin B12 pre-treatment, regardless of dosage, one day prior to PTZ injection experienced simple less aggressive twitches, which are twitches of less duration and violent contractions, while the majority of samples under those groups that underwent vitamin B12 pre-treatment, regardless of dosage, two weeks prior to PTZ injection showed more of simple less aggressive twitches rather than tonic-clonic convulsions.

## Discussion

Neuroinﬂammation is widely known to occur during epileptogenesis; thus, targeting it has been considered a promising approach toward the development of treatment for epilepsy [2]. For the past decades, several studies have revealed that epileptic activities are associated with vitamin B12 deﬁciency, as proven by Sklar (1986) [18], which led to emerging evidence regarding the neuroprotective effects of vitamin B12, as found by Romano et al. (2014) [19] and Tamaddonfard et al. (2012) [20]. This study provided results that were nearly consistent with other literatures in which vitamin B12 pre-treatment has shown an antiepileptic effect against PTZ-induced convulsions. The main ﬁndings of this study are as follows: (1) the latency period to convulsion increased after vitamin B12 pre-treatment, (2) the duration period of convulsion decreased after vitamin B12 pre-treatment, (3) the percentage of rats experiencing convulsion decreased after vitamin B12 pre-treatment, and (4) rats with vitamin B12 pre-treatment experienced simple twitching convulsion more rather than generalized tonic-clonic convulsion. In most animal experimental models, PTZ kindling is commonly used to demonstrate the effect of antiepileptic drugs against epileptogenesis [21]. According to Morimoto et al. (2004) [21], kindling refers to the stimulation of the brain, either chemically or electrically, that leads to the lowering of the seizure threshold allowing seizure occurrence. The generally recognized mechanism for PTZ-induced seizure is the noncompetitive antagonism of the gamma-aminobutyric acid (GABA)-A receptor complex, which suppresses the function of the inhibitory synapses [22].

In this study, the latency to PTZ-induced convulsion signiﬁcantly increased, while the duration signiﬁcantly decreased after vitamin B12 pre-treatment, regardless of the dosage and pre-treatment period. Several previous studies have demonstrated the beneficial effects of vitamin B12 in controlling seizures attacks, such as Empanfarast et al. (2017) [23], Taskıran et al. (2018) [24], and Filiz et al. (2021) [1]. However, our experimental protocol is different than what was used in these reports. In Empanfarast et al.’s (2017) [23] experiment, vitamin B12 was administered as intracortical microinjections. Taskıran et al.’s (2018) [24] protocol consisted of multiple intraperitoneal injections of vitamin B12 for a week before testing the animals with PTZ injections, while Filiz et al.’s (2021) [1] protocol was to affect kindling procedures, which consisted of subthreshold convulsion doses of PTZ combined with vitamin B12 administration for about a month. Our experiment was aiming to confirm that a single vitamin B12 injection can modulate the convulsive effect of PTZ.

In addition, detailed information about the onset, duration, severity, and probability of seizure induction was examined. Aside from vitamin B12, some compounds with potential antiepileptic effects were also explored in PTZ-administered rat models. Karabulut et al. (2021) [2] demonstrated that thiamine or vitamin B1 has effectively suppressed the PTZ-induced epileptogenesis activities among rat models (P<0.05) in which the 50 mg/kg dosage of thiamine was found more effective in reducing the said activities (P<0.05). While Mehla et al. (2010) [25] used curcumin to delay the development of PTZ kindling among rats in which 300 mg/kg was found to show a signiﬁcant increase in latency period. Focusing on the antiepileptic effects of vitamin B12, it has been documented that several epileptic activities, such as seizures, are commonly associated to vitamin B12 deﬁciency [15]. According to Hunt et al. (2014) [26], the main functions of vitamin B12 include the methylation processes related to DNA and cell metabolism. It affects the nervous system including its neuroprotective and neurotrophic actions, speciﬁcally in promoting growth and repair of nervous tissues during neuron injuries [1,26]. Thus, vitamin B12 deﬁciency might result in some serious neurological impairment. Although not clearly established, vitamin B12 level at 200 ng/L would be considered sensitive in diagnosing patients with such deﬁciency at 97% [26].

This study also determined the type of convulsion manifested by the rat models, either simple twitches or tonic-clonic. The results revealed that vitamin B12 pre-treatment favored the occurrence of simple less aggressive twitches rather than tonic-clonic convulsions, in which such ﬁndings were generally observed during a shorter PTZ and vitamin B12 injection gap time. In the study conducted by Filiz et al. (2021) [1], the behavioral seizure of PTZ-induced rat model was also monitored using the Racine scale in which simple twitching corresponded to low seizure effect while tonic-clonic seizures corresponded to the second highest seizure effect next to lethal seizures. The results revealed that vitamin B12 has shown protection against PTZ kindling in rats wherein oxidative stress and other neurological impairment were reduced. Similarly, Karabulut et al. (2022) [2] used the nearly equal ranking from simple twitches to clonic and lethal seizures in PTZ-induced rat models. Using thiamine as the antiepileptic component, the results revealed that seizures causing neurological and cognitive impairment have been alleviated. Relating to this, Lubana et al. (2015) [15] have demonstrated through a case report that vitamin B12 deﬁciency, along with high folate level, caused generalized tonic-clonic seizure in a human patient. These findings have been supported by the reduction of seizure episodes after vitamin B12 supplementation and folate normalization. As stated in their report, the deﬁciency in vitamin B12 hinders the methionine generation, which is relevant to the synthesis of myelin and neurotransmitters. In addition, deﬁciency leads to methylmalonic acid formation, a myelin destabilizer.

This study provided valuable insights into the effect of vitamin B12 on epileptogenesis in rats; however, there are still some limitations to be considered. Although PTZ is simple, it is considered laborious and time-consuming. In addition, PTZ kindling models have limitations regarding the analysis of seizure initiation and propagation patterns, which was also stated by Singh et al. (2021) [27]. Also, the trend on the effect of increasing the vitamin B12 dosage and pre-treatment period to the latency to and duration of convulsion would not be deﬁnite because this study worked at two data points each only. Lastly, the use of 100% female population may allow the results to be conclusive among female models only. Other factors affecting the seizure results, such as the device used and the expertise of the evaluators, were not reported or controlled.

## Conclusions

This study provided evidence that vitamin B12 pre-treatment can reduce seizures among PTZ-induced rat models. In general, the latency to convulsion has increased after pre-treatment, while the duration of convulsion has decreased. In addition, simple twitching seizures were more likely to occur than tonic-clonic seizures after the said pre-treatment. These findings then suggest that vitamin B12 is a potential strategy and treatment for epilepsy and other related epileptogenesis activities.
